# 

**Published:** 1896-01-25

**Authors:** 


CONTENTS.

The Limits of Medical Competition - - - - - 275

Coroners and Practitioners - - - - - - - 276

The Use of Sedatives in Phthisis. By SOLOMON C. SMITH, M.D., M.R.C.P. - - - - - - - - - - - 277

The Mechanism of Motion - - - - - - - - 278

Annotations.—The Whitechapel Guardians and the Local Doctors—The Evolution of the Winning Races—Milk a Vehicle of Disease - - - - - - - - - - - - 279

Notes and News - - - - - - - - - - - - 280

Scraps and Gleanings - - - - - - - - - - 289

The Book World of Medicine and Science - - - - 289

Medical Progress and Hospital Clinics—

Hæmorrhoids: Causes and Symptoms - - - - - 281

PROGRESS IN MEDICINE.—Diseases of the Lungs and Pleuræ (continued) - - - - - - - - - - - 282

PROGRESS IN SURGERY.—Head Injuries - - - - - 283

THERAPEUTIC NOTES—Morphine and Opium - - - - 284

BRITISH BALNEOLOGICAL AND CLIMATOLOGICAL SOCIETY - - 286

The Institutional Workshop—

HOSPITAL CONSTRUCTION - - - - - - - - 287

NEW HOSPITAL FOR BURTON-ON-TRENT - - - - - - 288

PRACTICAL POINTS - - - - - - - - - - - 288

EXTRA SUPPLEMENT.—THE NURSING MIRROR.

News from the Nursing World.—The late Prince Henry of Battenberg—Overtraining—After-care of the Insane—The Registration of Nurses—A Question of Age—A Nurses' Home at Sheffield—Distribution of Certificates at Sunderland—Health Missioning in Berlin—The Serious Charge against Three Great Hospitals—A Loved and Valued Nurse—How Nurses Catch Typhoid—Helsingfors Hospital—Short Items - cxxxvii

Lectures on Nursing. VI.—General Conduct - - - cxxxix

The Evolution of the Thermometer - - - - - cxl

Presentations - - - - - - - - - - - - cxl

The Midwife and the Midwifery Nurse - - - - cxli

Where to Go - - - - - - - - - - - - cxlii

Private Nursing - - - - - - - - - - - cxliii

Christmas Entertainments - - - - - - - - cxliii

Ophthalmic Hospital, Moorfields - - - - - - cxliv

Matrons and "Private Practice" (!) - - - - - cxliv

Funeral of an Army Nurse with Military Honours - cxliv

Everybody's Opinion - - - - - - - - - - cxlv

For Reading to the Sick - - - - - - - - - cxlvi

Notes and Queries - - - - - - - - - - - cxlvi

